# Red Blood Cell 2,3-Diphosphoglycerate Decreases in Response to a 30 km Time Trial Under Hypoxia in Cyclists

**DOI:** 10.3389/fphys.2021.670977

**Published:** 2021-06-15

**Authors:** Kamila Płoszczyca, Miłosz Czuba, Małgorzata Chalimoniuk, Robert Gajda, Marcin Baranowski

**Affiliations:** ^1^Department of Kinesiology, Institute of Sport – National Research Institute, Warsaw, Poland; ^2^Department of Physical Education and Health in Biala Podlaska, Józef Piłsudski University of Physical Education in Warsaw, Biala Podlaska, Poland; ^3^Center for Sports Cardiology, Gajda-Med Medical Center in Pułtusk, Pułtusk, Poland; ^4^Department of Physiology, Medical University of Bialystok, Bialystok, Poland

**Keywords:** hypoxia, hemoglobin oxygen affinity, oxygen dissociation curve, acid–base balance, acidosis, athletes, 2,3-diphosphoglycerate

## Abstract

Red blood cell 2,3-diphosphoglycerate (2,3-DPG) is one of the factors of rightward-shifted oxygen dissociation curves and decrease of Hb-O_2_ affinity. The reduction of Hb-O_2_ affinity is beneficial to O_2_ unloading at the tissue level. In the current literature, there are no studies about the changes in 2,3-DPG level following acute exercise in moderate hypoxia in athletes. For this reason, the aim of this study was to analyze the effect of prolonged intense exercise under normoxic and hypoxic conditions on 2,3-DPG level in cyclists. Fourteen male trained cyclists performed a simulation of a 30 km time trial (TT) in normoxia and normobaric hypoxia (FiO_2_ = 16.5%, ~2,000 m). During the TT, the following variables were measured: power, blood oxygen saturation (SpO_2_), and heart rate (HR). Before and immediately after exercise, the blood level of 2,3-DPG and acid–base equilibrium were determined. The results showed that the mean SpO_2_ during TT in hypoxia was 8% lower than in normoxia. The reduction of SpO_2_ in hypoxia resulted in a decrease of average power by 9.6% (*p* < 0.001) and an increase in the 30 km TT completion time by 3.8% (*p* < 0.01) compared to normoxia. The exercise in hypoxia caused a significant (*p* < 0.001) decrease in 2,3-DPG level by 17.6%. After exercise in normoxia, a downward trend of 2,3-DPG level was also observed, but this effect was not statistically significant. The analysis also revealed that changes of acid–base balance were significantly larger (*p* < 0.05) after exercise in hypoxia than in normoxia. In conclusion, intense exercise in hypoxic conditions leads to a decrease in 2,3-DPG concentration, primarily due to exercise-induced acidosis.

## Introduction

Oxygen transport to tissues is the primary factor determining the maximal oxygen uptake (VO_2max_) and aerobic exercise capacity of athletes (Wagner, 1996). Transport of O_2_ from the inspiratory air to tissues is dependent on several steps: ventilation, diffusion of O_2_ into red blood cells (RBCs) and its binding by hemoglobin (Hb), blood flow, and diffusion of O_2_ from the RBC to the mitochondria inside the cells (Mairbäurl, 1994; Wagner, 1996). One stage in this pathway is O_2_ transport *via* Hb. Improvements at this step can occur through an increase in the concentration of Hb per unit volume of blood and/or by the changes of Hb-O_2_ affinity. The increase in Hb level is a slow response that can be obtained from long-term adaptation to training or hypoxia (Pugh, 1964; Wehrlin et al., 2006; Schmidt and Prommer, 2008; Płoszczyca et al., 2018). By contrast, the regulation of Hb-O_2_ affinity is a fast process that occurs when RBC passes through capillaries, where they are exposed to changes in temperature, H^+^, and CO_2_ (Mairbäurl, 2013). Additionally, within the RBC, oxygen affinity is determined by various salts, including Cl^−^, ATP, and 2,3-diphosphoglycerate (2,3-DPG; Winslow, 2007). The increase of Hb-O_2_ affinity facilitates arterial O_2_ loading of Hb in the lung. The decrease of Hb-O_2_ affinity is beneficial to O_2_ unloading at the tissue level (e.g., in muscle tissue), and it is associated with shifting of the oxygen dissociation curve (ODC) to the right (Mairbäurl, 2013). The improvement in oxygen delivery resulting from left or right ODC shifts may be especially important during exercise in a hypoxic environment (Dempsey, 2020).

One of the factors of rightward-shifted ODC is an increase in 2,3-DPG concentration (Benesch and Benesch, 1967). 2,3-DPG is an intermediate metabolite in the Luebering–Rapoport glycolytic pathway. It is synthesized in RBC from 1,3-diphosphoglycerate (1,3-DPG) in the reaction catalyzed by diphosphoglycerate mutase. 2,3-DPG acts as a regulator of the allosteric properties of hemoglobin in the RBC. When 2,3-DPG is bound to hemoglobin, it stabilizes the T-state conformation and decreases hemoglobin affinity for oxygen (Benesch and Benesch, 1967; Brewer, 1974). The 2,3-DPG concentration may change in disease states (e.g., anemia, chronic obstructive lung disease, Parkinson’s disease, and cystic fibrosis; Alevizos and Stefanis, 1976; Acharya and Grimes, 1986; Illuchev et al., 1993; Böning et al., 2014) and under the influence of environmental conditions (Koistinen et al., 2000; Savourey et al., 2004). It has been shown that the increase in 2,3-DPG level is a positive adaptation following endurance training lasting several weeks or months (Böswart et al., 1980; Braumann et al., 1982; Hasibeder et al., 1987; Schmidt et al., 1988) and as a result of exposure to hypoxia (Lenfant et al., 1968; Mairbäurl et al., 1986b; Sutton et al., 1988). Acute hypoxic exposure causes hyperventilation and increases CO_2_ removal, leading to respiratory alkalosis and an increase in blood pH (Lühker et al., 2017). An increase in pH stimulates glycolysis, which contributes to an increase in the concentration of 2,3-DPG (Duhm and Gerlach, 1971).

The increase in 2,3-DPG concentration and the decrease of Hb-O_2_ affinity potentially should also be a positive change facilitating O_2_ unloading in muscles during exercise, especially when an imbalance between the O_2_ supply and demand is enhanced by a hypoxic environment. However, in a recent study, Dominelli et al. (2020) found that high-affinity hemoglobin mitigated the decline in maximal aerobic capacity and preserved pulmonary gas exchange during an incremental exercise test to exhaustion in hypoxia (FiO_2_ = 15%, ~2,500 m). These results indicated that a shift of the ODC to the left, not to the right, plays a positive role in the adaptation to exercise in hypoxic conditions. In a study conducted by Dominelli et al. (2020), the 2,3-DPG concentration was not measured, so the direction of the changes in 2,3-DPG level during intense exercise under hypoxia is unknown.

Previous studies on the effects of acute exercise in normoxia on 2,3-DPG level have shown conflicting results. Some authors have found an increase (Taunton et al., 1974; Remes et al., 1979; Meen et al., 1981), whereas others have found a reduction (Taunton et al., 1974; Ramsey and Pipoly, 1979) or no changes (Ricci et al., 1988; Spodaryk and Żołądź, 1998) in 2,3-DPG concentration after exercise in normoxia. The main reason for the discrepancies in results seems to be methodological differences, including the different intensity of the applied exercise and the participants’ sports level (Kuński and Sztobryn, 1976; Remes et al., 1979; Meen et al., 1981; Mairbäurl et al., 1986a).

Elevated 2,3-DPG concentrations may have beneficial effects on exercise capacity under normoxia (Remes et al., 1979; Cade et al., 1984). For this purpose, phosphate salt supplementation is used in endurance sports (cycling and triathlon; Buck et al., 2013). Theoretically, the benefits of such supplementation may also occur during intense exercise performed under hypoxia. The starting point for the study of ergogenic effects of phosphate salt supplementation is to investigate changes in 2,3-DPG concentration during exercise under hypoxia. In the current literature, there are no studies about the changes in 2,3-DPG level following acute exercise in moderate hypoxia in athletes. For this reason, the aim of the present study was to analyze the effect of a 30 km cycling time trial in normoxia and normobaric hypoxia (FiO_2_ = 16.5%, ~2,000 m) on RBC 2,3-DPG level in cyclists.

## Materials and Methods

### Participants

Fourteen male trained cyclists were recruited for this study (aged 25.9 ± 8.5 years; body height 179.6 ± 5.4 cm; body mass 69.6 ± 5.9 kg; fat content (%) 9.2 ± 2.2%; and VO_2max_ 61.1 ± 2.9 ml∙kg^−1^∙min^−1^). All athletes had competition experience and were familiar with our laboratory testing procedures. All participants had recently undergone medical examinations, without any contraindications that would exclude them from the study. The participants provided their written voluntary informed consent before participation. The research project was conducted according to the Helsinki Declaration and was approved (no. 10/2019, approval date: November 14, 2019) by the Ethics Committee for Scientific Research at the Jerzy Kukuczka Academy of Physical Education in Katowice, Poland.

### Experimental Design

The subjects were tested on two randomized occasions, separated by 7 days, in normoxic and hypoxic conditions (FiO_2_ = 16.5%, equivalent to 2,000 m). Study participants were allocated to conditions using a computer-generated randomized list (Urbaniak and Plous, 2013). On each occasion, the participants performed a simulation of a 30 km time trial in a laboratory. The most important consideration when choosing exercise tests is a strong relationship between competitive performance and performance in the test (Paton and Hopkins, 2001). We chose the laboratory simulation of 30 km time trial because this trial reflects the effort of an individual time trial in road cycling. Furthermore, the reproducibility of mean power and time to completion for laboratory cycling time trials is well established, with the reported CV of 1.9–3.6% (Smith et al., 2001; Laursen et al., 2003; Sporer and McKenzie, 2007; Zavorsky et al., 2007).

The subjects were blinded to exercise conditions. During TT, participants only had information available about the elapsed distance, cadence, and route profile. They had no information on power generated, time, current HR, SpO_2,_ and FiO_2_. After the experiment, we calculated the blinding index (BI) using the method proposed by Bang et al. (2004). The blinding index was −0.07 (95% CI, −0.33–0.18) in the hypoxia and −0.14 (95% CI, −0.49–0.20) in the normoxia, which suggests the success of blinding (values of BI sufficiently close to 0). Hypoxic conditions in the laboratory were created using a normobaric hypoxia system (AirZone 25, Air Sport, Poland), and the size of the hypoxic chamber was 45 m^2^. Throughout the experiment, for 2 days prior to each test series, participants stayed at the camp and consumed the same meals (40 kcal/kg of body weight per day, 50% carbohydrates, 20% proteins, and 30% fats). The athletes did not carry out strenuous exercise for 48 h prior to the time trial. The athletes were also instructed to avoid caffeine intake for 24 h prior to each test.

### Testing Protocol

On each test series, before breakfast, body mass and body composition were evaluated using the electrical impedance technique (Inbody 570, InBody Co., Seoul, Korea). Next, 2 h after a light breakfast, the simulated 30 km individual time trial in mountainous terrain was performed. Before TT, subjects performed a 15-min warm-up under the conditions in which the TT was performed. The warm-up was performed according to the individual preferences of the athletes. In hypoxic conditions, the warm-up and TT were conducted after 15 min of passive exposure to hypoxia. The exercise was performed on athletes’ personal bicycles connected to an electromagnetic bicycle trainer (Cyclus 2, RBM Elektronik-Automation GmbH, Leipzig, Germany). During the time trial, continuous power measurement was carried out. Additionally, at rest and after each 10 km of the time trial (10, 20, and 30 km), the blood oxygen saturation (SpO_2_) and heart rate (HR) were measured (WristOx2, Nonin Medical Inc., Plymouth, United States). During the test, subjects were allowed to consume water ad libitum. During all test series, the atmospheric conditions in regard to temperature (19°C), humidity (50%), concentration of carbon dioxide (700–800 ppm), and concentration of oxygen (FiO_2_ = 16.5%) were controlled and held constant to increase the reliability of the investigations. The time of day and the order of participants were also recorded, which was the same for all participants in both series of testing. All the participants followed the same 7-day training routines with individually adjusted intensity zones and 2 days of rest before each test series. Training load was recorded using power meters (Vector, Garmin). It was calculated after each training session and archived using WKO+ 4.0 software (TrainingPeaks, United States).

Before the exercise and immediately after its completion, the blood samples from the antecubital vein were collected to determine 2,3-DPG concentration and fingertip capillary blood samples were drawn for the assessment of lactate (LA) level and acid–base equilibrium (Cobas b 123 POC system, Roche Diagnostics GmbH, Mannheim, Germany).

### Determination of 2,3-DPG

To prepare samples for 2,3-DPG measurement, 2 ml of venous blood in heparinized tubes was collected, placed immediately on ice, deproteinized with 0.6 M perchloric acid (Sigma-Aldrich, Saint Louis, MO, United States) to lyse RBC, and neutralized with 2.5 M potassium carbonate (Sigma-Aldrich, Saint Louis, MO, United States). The supernatant was kept for at least 60 min in an ice bath and centrifuged at 3,000 × *g* for 10 min. The supernatant was stored at 28°C, and 2,3-DPG levels were measured using the Roche diagnostic kit (no. 10148334001). The Roche 2,3-DPG assay is based on enzymatic cleavage of 2,3-DPG, and oxidation of nicotinamide adenine dinucleotide recorded by spectrophotometry. The 2,3-DPG assays were performed in three batches and in the range of 0.02–0.15 μmol. Concentration of 2,3-DPG was calculated according to the procedure proposed by the manufacturer. The 2,3-DPG levels were normalized to the corresponding hematocrit value from the same sample. Since the concentration of 2,3-DPG rapidly decreases during storage (Hamasaki and Yamamoto, 2000; Llohn et al., 2005), the procedure for determining the 2,3-DPG level was performed immediately after taking the blood samples. Determination of 2,3-DPG level was carried out in duplicate on each sample. The reliability of 2,3-DPG measurement was evaluated based on the coefficient of variation (CV) using the test–retest method (Atkinson and Nevill, 1998). CV for 2,3-DPG was between 0.30 and 0.76%, which indicates that these measurements are characterized by a high degree of reliability.

### Statistical Analysis

The results of the study were analyzed using the StatSoft Statistica 13.0 software. The results were presented as arithmetic means (x) ± standard deviations (SD). The statistical significance was set at *p* < 0.05. Prior to all statistical analyses, normality of the distribution of variables was checked using the Shapiro–Wilk test. The comparisons in 2,3-DPG concentrations and SpO_2_ level were assessed by ANOVA for repeated measures (condition × time). When significant differences were found, the *post-hoc* Tukey’s test was used. The paired samples *t*-test was used to determine the significance of differences in acid–base balance and exercise performance level. The relationships between exercise-induced changes in 2,3-DPG concentration and sports level of participants, expressed as average relative power (W/kg) during time trial, were analyzed using Pearson’s correlation coefficient. Effect sizes (ESs) were calculated from standardized differences (Cohen’s *d* units). Threshold values for Cohen ES statistics were considered to be small (0.20–0.60), moderate (0.60–1.20), large (1.20–2.0), very large (2.0–4.0), or extremely large (>4.0; Hopkins et al., 2009).

## Results

### Time Trial Performance and SpO_2_

The paired samples *t*-test showed that average absolute power (*P*_avg_) during the 30 km TT was 9.6% lower (*t* = 8.64, *p* < 0.001, and *d* = 0.91) in hypoxia compared to normoxia (230 ± 22 W vs. 252 ± 26 W, respectively). The relative *P*_avg_ was also lower in hypoxia than in normoxia (*t* = 8.75, *p* < 0.001, and *d* = 1.14; 3.63 ± 0.30 W vs. 3.31 ± 0.26 W, respectively). The completion time of the 30 km TT was 3.8% longer (*t* = −3.83, *p* < 0.01, and *d* = 0.76) under hypoxic than normoxic conditions (3,834 ± 196 s vs. 3690 ± 182 s, respectively).

**Figure 1:**
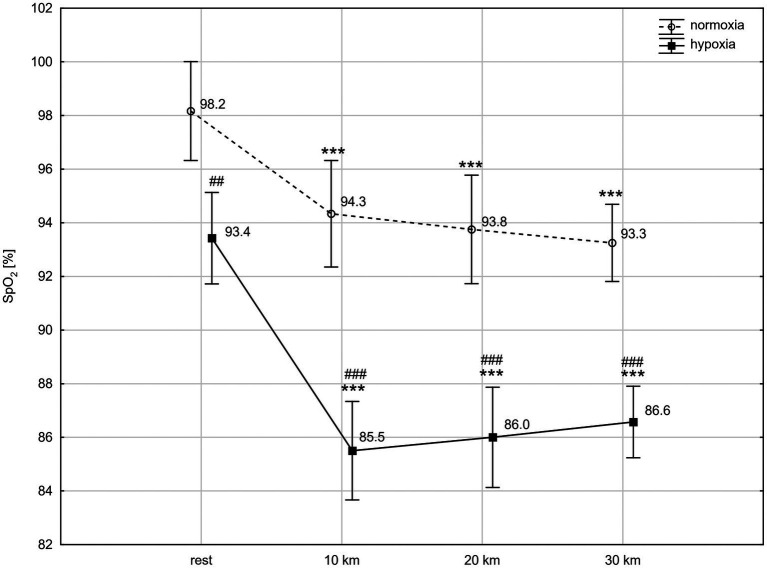
Blood oxygen saturation (SpO_2_) levels at rest and during 30 km time trial in normoxia and hypoxia. All values shown as mean ± SD; ^***^p < 0.001 – significant differences between rest and 10 km, 20 km, and 30 km time trial in the same conditions; ^##^*p* < 0.01, ^###^*p* < 0.001 – significant differences between N and H at the same measuring point.

ANOVA with repeated measures showed a significant interaction (condition × time) for SpO_2_ level (*F* = 4.60; *p* < 0.01) and a main time effect for HR (*F* = 157.20; *p* < 0.001). The *post-hoc* Tukey’s test revealed that SpO_2_ was significantly lower in hypoxia than in normoxia, both at rest (*p* < 0.01) and during the TT (*p* < 0.001). The exercise caused a significant reduction (*p* < 0.001) in SpO_2_ between rest and the tenth km of the TT in both conditions, by 4.0% in normoxia (*d* = 1.45) and by 8.5% in hypoxia (*d* = 2.02). Next, at the twentieth km and thirtieth km of the TT, SpO_2_ did not change significantly compared to the level reached at the tenth km, but remained significantly lower than at rest (Figure 1). HR significantly (*p* < 0.001) increased after the tenth km of the TT in both conditions, by 53% in normoxia (*d* = 3.23) and by 44% in hypoxia (*d* = 2.96). HR remained significantly higher up to the thirtieth km of the TT compared to rest values. Conditions did not differentiate HR at rest and during TT (HR at rest: 115 ± 25 bpm vs. 121 ± 22 bpm, at tenth km: 176 ± 9 bpm vs. 174 ± 11 bpm, at twentieth km: 177 ± 8 bpm vs. 175 ± 13 bpm, at thirtieth km: 179 ± 10 bpm vs. 180 ± 12 bpm, normoxia vs. hypoxia, respectively).

### Biochemical Analysis

**Figure 2:**
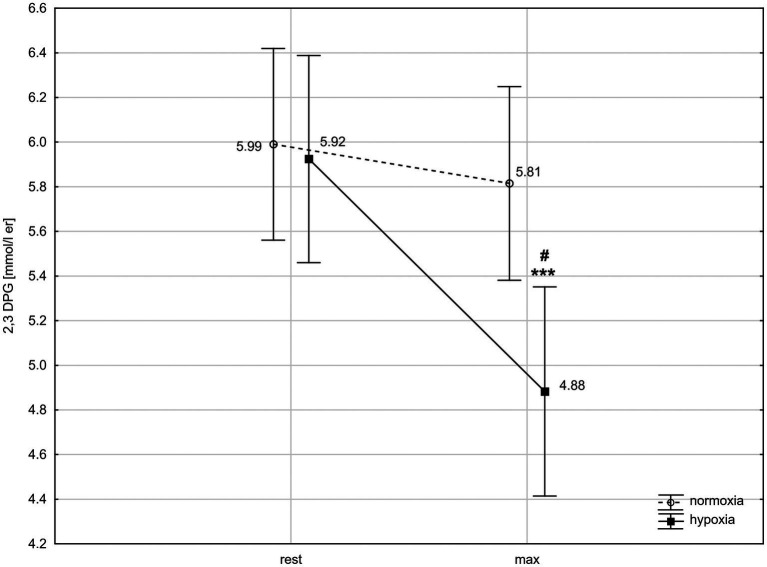
2,3-Diphosphoglycerate (2,3-DPG) levels in red blood cells before and after 30 km time trial in normoxia and hypoxia. All values shown as mean ± SD; rest – before time trial; max – immediately after time trial; ^***^p < 0.001 – significant difference between rest and max in the same conditions; ^#^*p* < 0.05 – significant difference between N and H at the same measuring point.

ANOVA with repeated measures showed a significant interaction (condition × time) for 2,3-DPG concentration (*F* = 9.76, *p* < 0.01). The *post-hoc* Tukey’s test revealed that 2,3-DPG level decreased significantly (*p* < 0.001; *d* = 1.22) by 17.6% after exercise in hypoxia but did not change significantly after exercise in normoxia. The 2,3-DPG level after the 30 km TT in hypoxia was significantly (*p* < 0.05; *d* = 1.18) lower than in normoxia (Figure 2).

**Figure 3:**
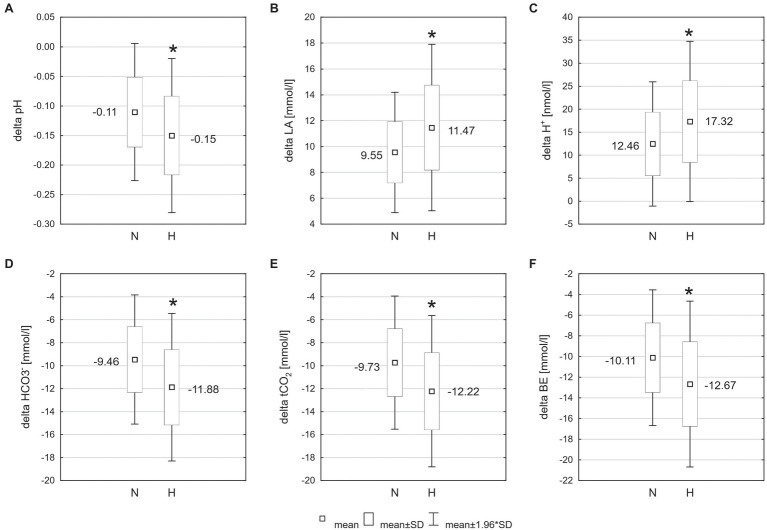
Changes of acid–base balance following 30 km time trial in normoxia (N) and hypoxia (H). ^*^p < 0.05 – significant differences between N and H; **(A)** delta pH – blood pH changes after exercise, **(B)** delta LA – change in blood lactate concentration, **(C)** delta H^+^ − change in hydrogen ion concentration, **(D)** ${\mathrm{HCO}}_{3}^{-}$ − change in bicarbonate concentration, **(E)** tCO_2_ – change in total carbon dioxide concentration, and **(F)** BE – change in base excess.

The exercise in normoxia caused a significant decrease in pH (*t* = 6.80, *p* < 0.001, and *d* = 2.39), ${\mathrm{HCO}}_{3}^{-}$ (*t* = 12.72, *p* < 0.001, and *d* = 4.30), tCO_2_ (*t* = 12.67, *p* < 0.001, and *d* = 4.31), and BE (*t* = 11.38, *p* < 0.001, and *d* = 3.86) levels and an increase in LA (*t* = −14.83, *p* < 0.001, and *d* = 5.36) and H^+^ (*t* = −6.71, *p* < 0.001, and *d* = 2.36) levels. After exercise in hypoxia, there was also a significant decrease in pH (*t* = 8.13, *p* < 0.001, and *d* = 2.92), ${\mathrm{HCO}}_{3}^{-}$ (*t* = 13.06, *p* < 0.001, and *d* = 5.24), tCO_2_ (*t* = 13.11, *p* < 0.001, and *d* = 5.28), and BE (*t* = 11.16, *p* < 0.001, and *d* = 4.28) levels and an increase in LA (*t* = −12.10, *p* < 0.001, and *d* = 5.34) and H^+^ (*t* = −6.76, *p* < 0.001, and *d* = 2.61) levels (Table 1). The analysis revealed that changes in acid–base balance were significantly larger (*p* < 0.05; *d* = 0.61–0.79) after exercise in hypoxia than following exercise in normoxia (Figure 3).

**Table 1:** Acid–base balance before and after 30 km time trial in normoxia (N) and hypoxia (H).

Variables	Conditions	Rest	Max
pH	N	7.41 ± 0.01	7.30 ± 0.06^***^
	H	7.40 ± 0.02	7.25 ± 0.07^***^
LA (mmol/l)	N	2.34 ± 0.54	11.99 ± 2.49^***^
	H	2.23 ± 0.54	13.70 ± 2.99^***^
H^+^ (nmol/l)	N	39.02 ± 1.07	50.89 ± 7.03^***^
	H	39.81 ± 2.09	57.13 ± 9.15^***^
${\mathrm{HCO}}_{3}^{-}$ (mmol/l)	N	24.56 ± 1.23	15.16 ± 2.83^***^
	H	25.10 ± 1.09	13.22 ± 3.01^***^
tCO_2_ (mmol/l)	N	25.78 ± 1.28	16.11 ± 2.90^***^
	H	26.36 ± 1.12	14.14 ± 3.07^***^
BE (mmol/l)	N	0.01 ± 1.08	−9.95 ± 3.48^***^
	H	0.29 ± 1.12	−12.38 ± 4.03^***^

All values shown as mean ± SD; rest – before time trial; max – immediately after time trial. pH, blood pH; LA, blood lactate concentration; H^+^, hydrogen ion concentration; ${\mathrm{HCO}}_{3}^{-},$ bicarbonate concentration; tCO_2_, total carbon dioxide concentration; and BE, base excess.

*** *p* < 0.001 – significant differences between rest and max.

No significant correlations were found between the changes in 2,3-DPG level (delta 2,3-DPG in mmol/l and %) and relative *P*_avg_ during the 30 km TT in both conditions.

## Discussion

The major factors of rightward-shifted ODC and facilitating the O_2_ unloading from Hb are decrease in pH and increase in temperature, CO_2_, and 2,3-DPG levels (Mairbäurl, 2013). Accordingly, we expected an increase in 2,3-DPG level during exercise, especially in hypoxic conditions, when oxygen availability is limited. Contrary to expectation, the results of our study showed that 2,3-DPG concentration significantly decreased after the 30 km cycling TT in hypoxia. After exercise in normoxia, a downward trend of 2,3-DPG level was also observed, but this effect was not statistically significant (Figure 2).

The results of prior research on the effect of exercise in normoxia on 2,3-DPG level are equivocal. Remes et al. (1979) observed an increase in 2,3-DPG concentration after a long-distance military march. Meen et al. (1981) reported that 2,3-DPG increased after 60 min of aerobic exercise and was unchanged or slightly reduced immediately after 6 min of intense exercise in trained subjects. Taunton et al. (1974) observed a decline in 2,3-DPG level after competition of a 10 mile road race in highly trained endurance athletes. In contrast, maximal exercise of the biathlon race (Ricci et al., 1988), long-distance running competition (Brodthagen et al., 1985), and incremental exercise test to exhaustion (Spodaryk and Żołądź, 1998) did not affect the 2,3-DPG level. On the basis of previous results, it appears that the reason for the discrepancy in 2,3-DPG response to exercise in normoxia is primarily the different intensity of the exercise. So far, the results in this regard remain inconsistent and it is not possible to clearly define the 2,3-DPG response to the exercise. Our results indicate that a downward trend in blood 2,3-DPG level can be expected following intense endurance exercise (above lactate threshold), which is consistent with results obtained by Taunton et al. (1974) and Brodthagen et al. (1985). After low-intensity endurance exercise, 2,3-DPG tends to increase (Remes et al., 1979; Meen et al., 1981). However, it should be noted that basal 2,3-DPG level and the 2,3-DPG response to exercise also depend on the sports level of the subjects (Kuński and Sztobryn, 1976; Remes et al., 1979).It was observed that athletes have a higher baseline 2,3-DPG level than untrained subjects (Taunton et al., 1974; Remes et al., 1979; Brodthagen et al., 1985; Ricci et al., 1988). There is a significant positive relationship between the resting concentration of 2,3-DPG and VO_2max_ (Remes et al., 1979; Mairbäurl et al., 1983; Czuba et al., 2009). It has been shown that 2,3-DPG concentration increases under the influence of exercise training (Braumann et al., 1982; Hasibeder et al., 1987; Hespel et al., 1988; Schmidt et al., 1988), which is mainly due to the stimulation of erythropoiesis and an increase in the number of young RBC, which are characterized by higher metabolic activity and an increased level of 2,3-DPG (Mairbäurl et al., 1983). It was also noted that in the subjects with a higher sport performance level, concentration of 2,3-DPG decreases after exercise, while it increases or is unchanged in subjects with lower fitness (Thomson et al., 1974; Kuński and Sztobryn, 1976; Remes et al., 1979). Our results showed no correlation between cycling performance level and the changes in 2,3-DPG level after exercise in both normoxia and hypoxia. However, it should be noted that in our study, the sports level of the participants was homogeneous. All athletes had at least several years of training and competition experience.

We suspect that the differences in 2,3-DPG response to exercise between well-trained and less-trained subjects are related to the most favorable adjustments in Hb-O_2_ affinity in order to optimize tissue oxygen supply. A high level of 2,3-DPG increases the Bohr effect on Hb-O_2_ affinity (Bauer, 1969) and is beneficial to O_2_ unloading in muscles during exercise. However, at the lung level, Hb-O_2_ affinity should be higher for better arterial O_2_ loading of Hb (Mairbäurl, 2013). Higher Hb-O_2_ affinity is favored by a higher pH, as well as a lower CO_2_ level and a lower temperature in the lungs compared to the working muscles (Mairbäurl, 1994). However, during intense exercise, normal values of Hb-O_2_ affinity are not fully restored, which leads to deterioration of arterial O_2_ loading of Hb and results in a reduction of hemoglobin saturation (Mairbäurl, 2013). Untrained individuals are usually not diffusion limited in the lungs, even during maximal exercise (Wagner, 2007). By contrast, in athletes with high cardiac output, arterial O_2_ loading of Hb is further hampered by diffusion limitation on account of the shortened pulmonary capillary RBC transit time (Dempsey and Wagner, 1999; Hopkins, 2006). The increase in 2,3-DPG level and a rightward shift of ODC, in the absence of a diffusion reserve, would further deteriorate the O_2_ loading of Hb in the alveoli in athletes. It is possible that for this reason, in well-trained individuals, 2,3-DPG level is regulated downward during intense exercise, which also happened in our study.

It is also likely that there is a certain threshold for a possible increase in 2,3-DPG level. It is known that 2,3-DPG synthesis is regulated *via* its own concentration (Mairbäurl, 1994). An increase in 2,3-DPG concentration competitively inhibits its substrate 1,3-DPG (Brown and Keith, 1993), decreases the red cell pH (Duhm and Gerlach, 1971), and reduces the activity of phosphofructokinase (PFK) and hexokinase (HK), the main glycolytic enzymes of RBC (Srivastava and Beutler, 1972; Brewer, 1974), which results in inhibition of 2,3-DPG synthesis. Perhaps, in athletes with a high baseline level of 2,3-DPG, this self-regulatory mechanism plays a significant role. However, this issue remains to be investigated further.

During hypoxic conditions, when the availability of oxygen is limited, there is a decrease in PaO_2_ and SpO_2_, which results in lowering of aerobic exercise performance (Lawler et al., 1988; Wehrlin and Hallen, 2006; Mollard et al., 2007; Faiss et al., 2014; Czuba et al., 2019; Piotrowicz et al., 2020). The decrease in aerobic performance is greater when the level of hypoxia is higher (Rusko et al., 2004; Piotrowicz et al., 2019). However, the magnitude of the decrease shows considerable individual variation (Chapman, 2013). Dempsey and Wagner (1999) indicated that each 1% decrement in SaO_2_ below the 95% level causes an ∼1–2% reduction in VO_2max_ (Dempsey and Wagner, 1999). The performance decrease from normoxia to hypoxia also manifests through the reduction of average power output during the time trial, about 4–5% at an altitude of 1,200–1,500 m to even nearly 20% at an altitude of 3,200 m (Amann et al., 2006; Clark et al., 2007; Weavil et al., 2015). In our study, mean SpO_2_ during the TT in hypoxia (2,000 m) was 86% and it was 8% lower than in normoxia. The reduction of SpO_2_ resulted in a lower *P*_avg_ (by 9.6%) and an increase in the 30 km TT completion time (by 3.8%). Similar results have been reported in earlier research (Amann et al., 2006; Clark et al., 2007; Saugy et al., 2016).

It would seem that the increase in the level of 2,3-DPG and facilitation of O_2_ unloading in muscles would be a favorable adaptive change during hypoxic exercise.The 2,3-DPG concentration increases as a result of acclimatization to altitude (Lenfant et al., 1968; Sutton et al., 1988). This increase is already observed during the first few hours of exposure to hypoxia (Savourey et al., 2004), and the changes persist with prolonged passive exposure or training under hypoxic conditions (Lenfant et al., 1968; Rörth et al., 1972; Mairbäurl et al., 1986b; Koistinen et al., 2000). This change is beneficial for the oxygen delivery to tissues during rest and moderate exercise at altitude up to 5,400 m. In contrast, at extreme altitude, the rightward-shifted ODC is a maladaptive response (Samaja et al., 1986) and the increase of Hb-O_2_ affinity is observed (West and Wagner, 1980). Early research suggested that shift of ODC to the right is also unfavorable during very heavy exercise (Samaja et al., 1986). In the latest study, Dominelli et al. (2020) indicated that the increased Hb-O_2_ affinity is a superior strategy for preserving exercise tolerance in acute hypoxia. In this case, an increase in 2,3-DPG level, as we expected, would not be advisable during intense exercise in hypoxic conditions.

We think that the change in 2,3-DPG concentration following the time trial was regulated mainly by factors related to the metabolic response to exercise. Exercise in hypoxia generated much more pronounced changes of acid–base balance and simultaneously a greater decline in 2,3-DPG level than exercise in normoxia. Based on our results and research by other authors, we suspect that exercise-produced acidosis was the primary factor causing the decrease in 2,3-DPG concentration during the time trial in hypoxia. Several previous studies have demonstrated a decrease in 2,3-DPG level following induced acidosis (Bellingham et al., 1971; Rapoport et al., 1977; Ramsey and Pipoly, 1979; Mairbäurl et al., 1986a; Hasibeder et al., 1987; Czuba et al., 2008). The extent of the decrease in 2,3-DPG level is related to the severity of the acidosis. Mairbäurl et al. (1986a) found that 2,3-DPG level declined during an incremental test to exhaustion when the acidosis was severe, but not when it was moderate. Bard and Teasdale (1979) reported that the reduction in blood pH by 0.010 units causes a simultaneous (4%) drop in 2,3-DPG concentration. In our study, we observed an ~18% decrease in 2,3-DPG level with a 0.150 decrease in pH, which indicates an ~1.2% decrease in 2,3-DPG level for every 0.010 decrease in blood pH. Interestingly, under normoxic conditions, when the pH drop was 0.110 units, 2.3-DPG concentration decreased by only 3.0% (not statistically significant). These results indicate that the change in 2,3-DPG level is not linear. A marked drop in 2,3-DPG should be expected when acidosis reaches a certain threshold (Mairbäurl et al., 1986a). Additionally, it has been suggested that the 2,3-DPG response to exercise may be affected by the duration of RBC exposure to the low pH (Spodaryk and Żołądź, 1998). When the time of exposure to acidification is too short (only a few minutes), the 2,3-DPG level is not decreased after exercise, despite the low pH (Taunton et al., 1974; Böswart et al., 1980; Meen et al., 1981).

The reduction in 2,3-DPG concentration due to acidosis is explained by two main mechanisms: (1) inhibition of glycolysis of RBC and (2) the changes in the activity of enzymes responsible for the synthesis and degradation of 2,3-DPG (Mairbäurl, 1994). A low pH reduces the rate of glycolysis by inhibition of PFK (Hollidge-Horvat et al., 1999), while contributing to the reduction of all glycolytic intermediates, including 2,3-DPG (Bonner et al., 1975; Brown and Keith, 1993). Moreover, acidosis inhibits 2,3-DPG mutase and activates 2,3-DPG phosphatase, the enzymes directly responsible for synthesis and degradation of 2,3-DPG, respectively (Rose, 1970; Rapoport et al., 1977).

Interestingly, in response to exercise and hypoxia, a number of other mechanisms are triggered, which potentially, in contrast to acidosis, should lead to an increase in 2,3-DPG level. The factors contributing to stimulation of 2,3-DPG production include hyperventilation (Brewer and Eaton, 1971; MacDonald, 1977), increases of catecholamine concentration (Remes et al., 1979; Katz et al., 1984), and increases in serum inorganic phosphate (Brewer, 1974; Czuba et al., 2009). Moreover, under hypoxic conditions, the desaturation of hemoglobin increases. Since deoxyhemoglobin binds 2,3-DPG with a greater affinity than oxyhemoglobin (Brewer and Eaton, 1971), the reduction of free 2,3-DPG by binding to Hb upon deoxygenation increases the rate of 2,3-DPG synthesis (Duhm and Gerlach, 1971). However, it seems that the above mechanisms are insufficient to counteract the inhibition of red cell glycolysis and reduction of 2,3-DPG synthesis resulting from increased acidosis during intense exercise.

## Study Limitations and Perspectives

In our study, the partial pressure of oxygen at which 50% of Hb is saturated with oxygen (p50) was not determined. Therefore, it is not known to what extent exercise in normoxia and hypoxia changed the Hb-O_2_ affinity and whether a greater decrease in 2,3-DPG level under hypoxic exercise had an effect on p50. These aspects should be considered in future research. It is also worth considering the comparison of passive hypoxic exposure with exercise of varying intensity under moderate hypoxic conditions in the context of changes in 2,3-DPG and p50 levels. Savourey et al. (2004) observed that 2,3-DPG increased significantly after 75 min of passive exposure to hypobaric hypoxia (4,500 m). In our study, hypoxic exposure duration (rest + intense exercise) was also ~75 min. In this period, the 2,3-DPG level decreased, probably as a result of exercise-induced acidosis. In our study, hypoxic level corresponded to an altitude of 2,000 m. It is of interest whether the 2,3-DPG level would increase after passive exposure to a simulated altitude of 2,000 m. Maybe a greater hypoxic stimulus is needed to stimulate 2,3-DPG production? And further, what effect would continuous aerobic exercise – during which metabolic acidosis does not occur but a hypoxic stimulus does – have on the 2,3-DPG concentration?

## Practical Application

In this study, we demonstrated that the 2,3-DPG concentration decreases after a simulated cycling time trial. The exercise test used reflects the effort involved in a road cycling competition. Therefore, we expect the 2,3-DPG response during the competition (time trial) to be similar to that obtained in our study. Since there is a relationship between the resting concentration of 2,3-DPG and VO_2max_ in normoxia (Remes et al., 1979; Mairbäurl et al., 1983; Czuba et al., 2009), we propose that future research should focus on analyzing whether an increase in basal 2,3-DPG level improves aerobic capacity under hypoxic conditions. Furthermore, an important issue from the point of view of coaches and athletes is whether supplementation with phosphate salts or other ergogenic aids may be beneficial in this regard?

## Conclusion

Moderate normobaric hypoxia (FiO_2_ = 16.5%, ~2,000 m) causes a decrease in SpO_2_ and a deterioration of 30 km cycling time trial performance by about 10% in trained cyclists. Intense exercise in hypoxic conditions leads to a significant decrease in the RBC 2,3-DPG concentration. Exercise-induced acidosis plays an essential role in the drop in 2,3-DPG level during intense exercise in hypoxia. The changes in the 2,3-DPG level during intense exercise in acute moderate hypoxia (2,000 m) are opposite to those reported by other authors during passive exposure to hypoxia. It is possible that the 2,3-DPG-induced rightward shift in ODC does not play a key role during exercise under hypoxic conditions, and shifting ODC to the left is a more favorable change. It is also likely that reduction of Hb-O_2_ affinity by a 2,3-DPG-dependent mechanism is less important when Hb-O_2_ affinity is mainly decreased by exercise-induced acidosis.

## Data Availability Statement

The raw data supporting the conclusions of this article will be made available by the authors, without undue reservation.

## Ethics Statement

The studies involving human participants were reviewed and approved by the Ethics Committee for Scientific Research at the Jerzy Kukuczka Academy of Physical Education in Katowice, Poland. The patients/participants provided their written informed consent to participate in this study.

## Author Contributions

KP and MCz: conceptualization, writing—review, and editing. KP, MCz, MCh, and MB: methodology. KP, MCz, MCh, RG, and MB: investigation. KP, MCh, and RG: database collection. KP: statistical analysis and writing – original draft. MCz: supervised the study. KP, MCz, and RG: funding acquisition. All authors contributed to the article and approved the submitted version.

### Conflict of Interest

The authors declare that the research was conducted in the absence of any commercial or financial relationships that could be construed as a potential conflict of interest.
